# Berta, an Open-Source, Modular AI Scribe for Clinical Documentation: Implementation Report

**DOI:** 10.2196/105385

**Published:** 2026-11-10

**Authors:** Samridhi Vaid, Mike Weldon, Jesse Dunn, Sacha Davis, Kevin Lonergan, Henry Li, Jeffrey Franc, Elda Dervishi, Mohamed Abdalla, Daniel C Baumgart, Jake Hayward, J Ross Mitchell

**Affiliations:** 1 Department of Medicine, Faculty of Medicine and Dentistry, University of Alberta, 5-134A Clinical Sciences Building, 11304 83 Avenue NW, Edmonton, AB, T6G 2B7, Canada, +1 780-492-7711; 2 Alberta Health Services, Edmonton, AB, Canada; 3 Department of Emergency Medicine, Faculty of Medicine and Dentistry, University of Alberta, Edmonton, AB, Canada; 4 Department of Computing Science, University of Alberta, Edmonton, AB, Canada; 5 Alberta Machine Intelligence Institute, Edmonton, AB, Canada

**Keywords:** artificial intelligence, AI, natural language processing, electronic health records, clinical documentation, medical informatics, ambient AI scribe, large language models, speech recognition software, emergency medicine, open-source software

## Abstract

**Background:**

Clinical documentation is a major contributor to physician workload and burnout, particularly in high-acuity settings such as emergency departments. Ambient AI scribes have emerged as a response, but commercial products cost US $99 to more than US $600 per physician per month, operate as opaque systems, and often process data on vendor-controlled infrastructure. These characteristics limit organizational control over data sovereignty, quality improvement, and clinical workflows and create barriers to adoption in resource-constrained settings.

**Objective:**

We aimed to develop an open-source, modular AI scribe platform that could be deployed entirely within institutional infrastructure, and to evaluate its real-world use across a provincial health system.

**Methods:**

We developed Berta, an open-source, modular platform that pairs a Next.js front end and a FastAPI back end with configurable automatic speech recognition and large language model components. A closed-source implementation developed in parallel with Berta was deployed at Alberta Health Services (AHS), Canada’s largest provincially integrated health system at the time of implementation, and operated within AHS’s existing Snowflake AI Data Cloud infrastructure. All clinical audio and text remained within the secure AHS environment. Use and session metadata were automatically recorded during routine clinical use by emergency physicians across AHS between November 2024 and July 2025, and physicians were emailed an optional survey. Operating costs were estimated from Snowflake service consumption over the same period.

**Implementation (Results):**

During the 9-month study period, 197 emergency physicians registered at 105 urban and rural facilities used the system, generating 21,548 clinical sessions and more than 2700 hours of audio. Monthly session volume grew from 675 sessions in November 2024 to 5463 in July 2025. Mean recorded session length was 7.6 minutes, and 42% (82/197) of users customized at least 1 documentation template. Operating costs averaged less than US $30 per physician per month, representing a 70% to 95% reduction in cost compared with commercial subscription prices; this comparison excludes personnel costs for development and maintenance. Survey respondents (69-73 per item) rated user-friendliness as good (median 5, IQR 4-6), reported a median time saving of 16 to 30 (IQR 0-15 to 46-60) minutes per shift, and rated documentation quality the lowest. After the pilot, access was opened to other departments, and by September 30, 2026, approximately 2000 users had registered.

**Conclusions:**

An in-house AI scribe can be deployed at provincial scale within existing health system infrastructure at a fraction of the operating cost of commercial alternatives while preserving data sovereignty and institutional control. The full cost of ownership and documentation quality require further evaluation. By releasing Berta publicly, we provide a reproducible foundation that health systems can adapt to evaluate and deploy AI documentation technology within their own secure environments.

## Introduction

### Context

Alberta Health Services (AHS) was, at the time of implementation, Canada’s largest provincially integrated health system, providing publicly funded care to approximately 5 million residents in urban centers and dispersed rural communities. Clinical documentation is recorded in Connect Care, a provincewide electronic health record (EHR) system (Epic Systems) used at more than 1000 sites and programs by more than 125,000 users [1]. The AI scribe described here was not integrated with Connect Care. The implementation was not part of a national program; rather, it progressed from a pilot conducted between November 2024 and July 2025 to an approved provincial scale-up.

### Problem Statement

The administrative burden of clinical documentation has become a defining challenge in modern health care. Physicians now spend approximately 2 hours on EHRs and desk work for every hour of direct patient care [2], with documentation that frequently extends into personal hours [2-4], a phenomenon known as “pajama time” [5]. Excessive documentation demands are consistently associated with physician burnout [6], reduced job satisfaction [6], and workforce attrition [7,8]. The consequences extend to patients, as time spent on documentation is time diverted from direct clinical care, communication, and shared decision-making [9]. These demands are particularly burdensome in high-acuity settings such as emergency departments, where the workforce is already under strain [10-12] and time-sensitive clinical decisions must compete with the requirement to produce prompt, thorough records.

### Similar Interventions

Human scribes were an early response to this burden: evaluations have reported reductions in documentation time [13], increased patient throughput [14], and improvements in patient and physician satisfaction [15,16]. Ambient AI scribes (systems that passively capture clinical conversations, transcribe speech, and generate structured documentation) have since emerged as a promising alternative [17]. These tools combine automatic speech recognition (ASR) with large language models (LLMs) to produce draft clinical notes that physicians can review and edit, reducing the cognitive and temporal costs of documentation. Pilot scribe projects involving AI scribes have become mainstream in recent years, with products from vendors such as Ambience Healthcare, Doximity, and Abridge now deployed in routine clinical practice [18].

However, the commercial landscape presents significant barriers to equitable adoption [19-21]. Subscription costs ranging from US $99 to more than US $600 per physician per month [22,23] place these tools beyond the reach of resource-constrained health systems, rural settings, and low- and middle-income countries. Commercial AI scribes operate as opaque systems [24] in which clinical data are often processed on vendor-controlled infrastructure, limiting institutional control over data governance, privacy compliance, and the secondary use of data for quality improvement or research [25,26]. Organizations cannot audit model behavior, customize outputs to local workflows, or retain generated data assets. This dependency introduces strategic risks, including pricing escalations, service discontinuation, and vendor lock-in. Some commercial products can be hosted within institutional environments, addressing data residency requirements; however, their models, prompts, and updates remain closed to independent audit, and per-physician licensing fees still apply. Berta was developed independently of these products as an open, modular alternative that can be deployed within an institution’s own infrastructure.

## Methods

### Aims and Objectives

We developed Berta (Alberta Health Services), an open-source, modular platform for AI-enabled clinical documentation. The system can be deployed entirely within institutional infrastructure, preserving data sovereignty while allowing organizations to customize and audit their own speech recognition and language model components. In partnership with AHS, a closed-source implementation developed alongside Berta was deployed within existing enterprise infrastructure. In this paper, we describe the system architecture, report on 9 months of operational use by emergency physicians, share lessons learned from the implementation, and release the codebase to provide a reproducible foundation for health systems deploying AI documentation technology in secure environments.

This was a descriptive implementation study. This report follows the iCHECK-DH (Guidelines and Checklist for the Reporting on Digital Health Implementations) guidelines [27] (Checklist 1). Key performance indicators were session counts, unique users, audio duration, template customization, monthly use, and operating cost per physician per month; clinical outcomes were outside the scope of the pilot.

### Co-Design and Development

Berta was co-designed by a team of 4 emergency physicians (MW, JH, HL, and JF), 2 machine learning engineers (SV and JD), 1 project analyst (KL), and 2 supervising professors (JRM and MA) using an iterative, user-centered development process. The clinical team used the AHS deployment in routine practice and provided structured feedback during weekly meetings, which supported ongoing updates to align the system with routine emergency department workflows. Core development took place between March 2024 and November 2024 and required approximately 13 person-months of engineering effort (provided by 1 full-time and 1 part-time machine learning engineer), supported by a 1-hour weekly meeting with the clinical team. Refinements validated during the AHS pilot were incorporated into the open-source Berta release.

### Technical Architecture

Berta comprises a Next.js (Vercel Inc) front end and a FastAPI [28] back end that exposes RESTful APIs for application logic, data processing, and integration (Figure 1).

**Figure 1.**
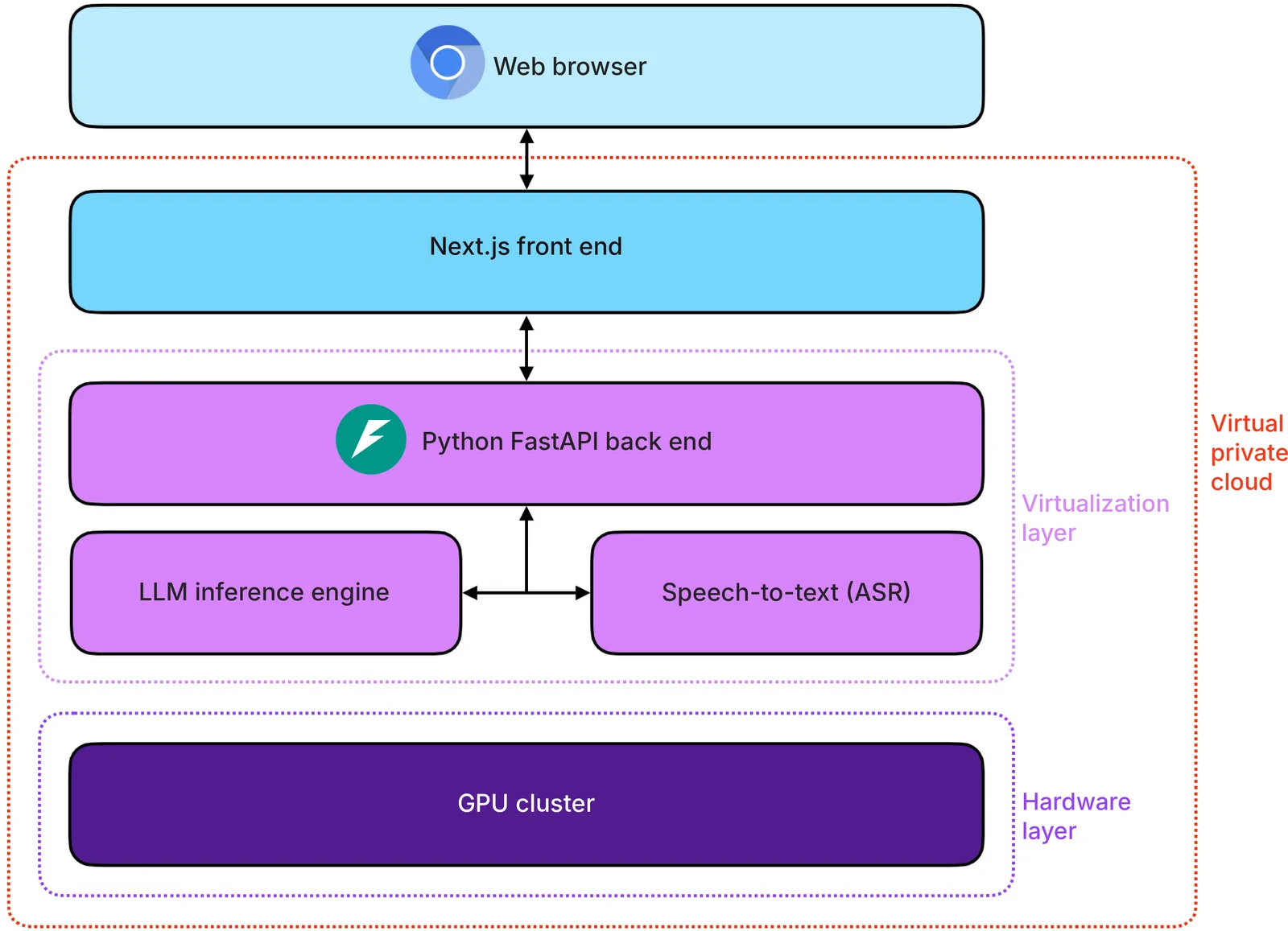
Berta system architecture. The platform features a multilayer design comprising a Next.js front end and a Python FastAPI back end coupled with a large language model (LLM) inference engine and a speech-to-text module for automatic speech recognition (ASR). Multiple inference engines are supported, including vLLM, Ollama, LM Studio, and any OpenAI application programming interface (API) compatible endpoint. Multiple speech-to-text modules are also supported, including WhisperX, Amazon Transcribe, NVIDIA Parakeet v2, and others. Berta supports on-premises and cloud-based graphics processing unit (GPU) acceleration and can be deployed securely within a virtual private cloud, with support for multiple user authentication systems. Icons are courtesy of Wikimedia Commons (Chromium) and the FastAPI project..

In routine use, clinicians create a session in a desktop or mobile browser (Figure 2) and record or upload audio from a patient encounter. The system transcribes speech with an ASR model and then uses an LLM to generate a structured draft clinical note from the transcript using configurable note templates (eg, full visit, narrative, and handover); users can also create and save custom templates. Clinicians review and edit the generated note before transferring it to the EHR by copy and paste.

The platform is modular with respect to its ASR and LLM components (Table 1). Deployments can run on a single workstation or on a graphics processing unit (GPU) server within a secure virtual private cloud or in enterprise cloud environments (eg, Snowflake AI Data Cloud [Snowflake Inc] and Amazon Web Services), enabling institution-controlled data governance. The Berta codebase is released under the Apache 2.0 license (Apache Software Foundation) [29].

**Figure 2.**
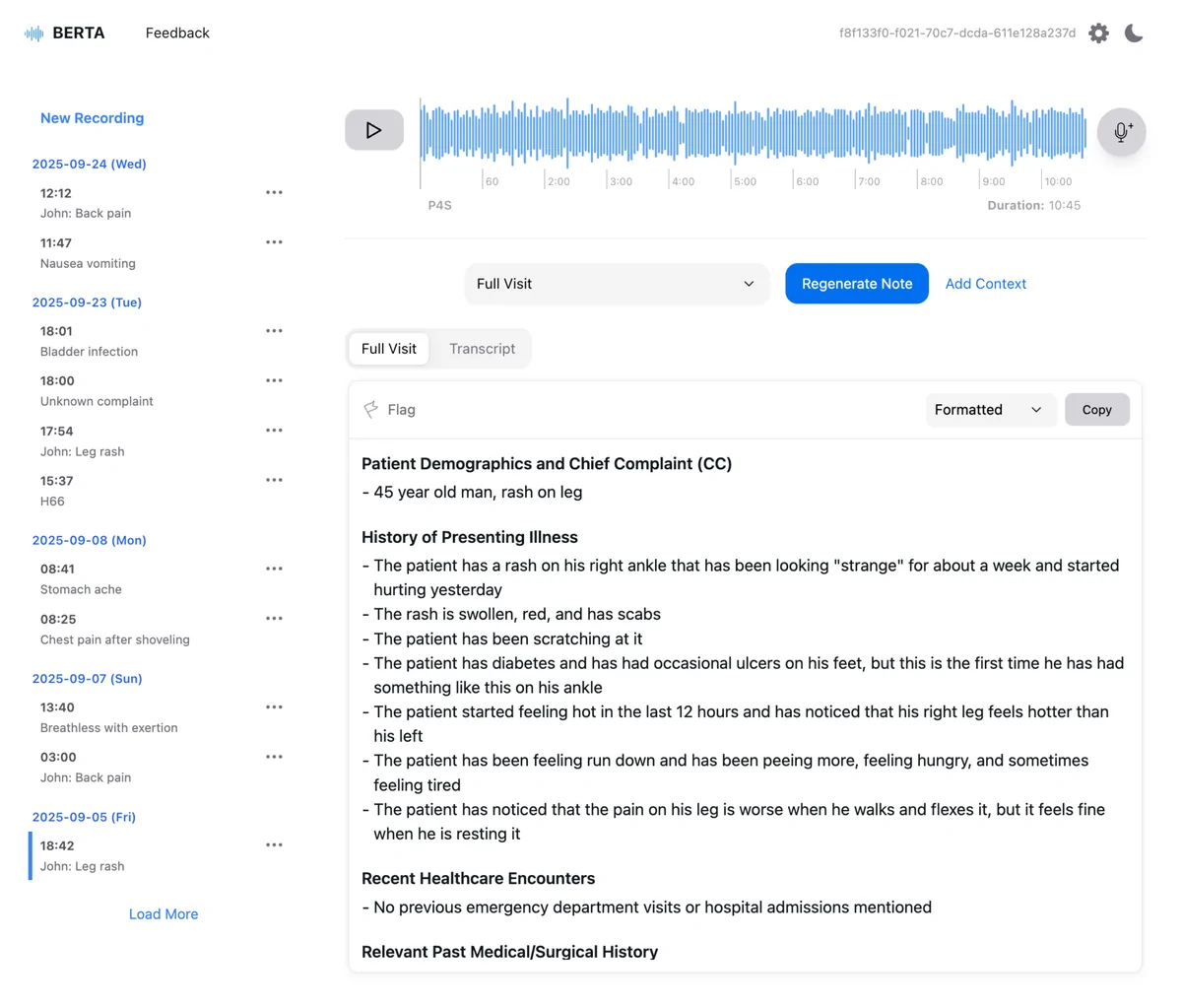
The Berta user interface. The left sidebar shows a chronological list of recorded sessions; the main area displays the audio waveform for the selected session and the automatically generated clinical note. All data shown are from simulated patient sessions; no real patient information is displayed.

**Table 1.** Supported automatic speech recognition (ASR) and large language model (LLM) back ends and deployment targets in the Berta platform.

Component	Local or on-premises options	Cloud or commercial options
ASR	WhisperX [30], OpenAI Whisper [31], and NVIDIA Parakeet via MLX [32-34]	Amazon Transcribe [35]
LLM inference engine	Ollama [36], vLLM [37], and LM Studio [38]	OpenAI API [39] and Amazon Bedrock [40]
Deployment target	Single workstation and on-premises GPU^a^ server	Virtual private cloud (eg, Snowflake AI Data Cloud or Amazon Web Services)

a GPU: graphics processing unit.

### Pilot Implementation

In November 2024, a closed-source implementation was deployed at AHS; internally, the deployment is known as “Jenkins” or the “AHS Digital Scribe.” Jenkins was developed in parallel with the open-source Berta codebase by overlapping teams; the 2 share design, functionality, and portions of the code but are maintained separately. The pilot ran within AHS’s Snowflake AI Data Cloud environment and used WhisperX [30] (OpenAI; an extension of OpenAI Whisper [31]) for transcription and a Snowflake-provisioned GPT-4o model (OpenAI) hosted in a private cloud [41] for note generation. All clinical audio and text remained within the AHS firewall and were not exported. Access was granted to individual physicians, not sites, after registration and brief online training. Adoption was voluntary and spread by word of mouth. After initial adoption by early users, physicians at additional emergency departments were invited in weekly waves between April and June 2025.

### Data Governance

Physicians were instructed to obtain patients’ verbal consent using a standard script stating that the tool records the conversation, that the physician reviews all output before it enters the chart, and that patients may decline or withdraw consent at any time without affecting their care. Physicians can delete recordings; 3677 (17%) of 21,548 pilot sessions were deleted. Retained recordings are held by AHS as the data custodian under the same provincial health information legislation that governs other clinical records and are kept for the duration of the project.

### Interoperability

The deployment was not integrated with Connect Care (Epic Systems Corp); physicians transferred generated notes by copy and paste, and no clinical terminology or data exchange standards (eg, Fast Healthcare Interoperability Resources) were used. Standards-based EHR integration is planned.

### Participating Entities

Berta was developed by a University of Alberta team in partnership with AHS, the provincial health authority, which owns and operates the deployed application (Jenkins).

### Costs

Operating costs per physician per month were estimated from Snowflake service consumption aggregated across web application and transcription container servers, LLM tokens, and storage. These estimates cover operating consumption only. They exclude development labor and ongoing personnel costs for maintenance, monitoring, cybersecurity, privacy review, and quality assurance. The project was supported by a 2-year Health Care Unburdened grant of CAD $1 million (CAD $1=US $0.73, at the time of the grant award, June 2024).

### Sustainability

Following the pilot, access to the AHS deployment was opened to other departments, thereby institutionalizing its use. Since deployment, maintenance and upgrades have been carried out by 1 developer, with additional support from existing AHS information technology staff. As these costs are absorbed within AHS operations, they were not tracked separately.

The open-source Berta platform is released under the Apache 2.0 license, so organizations that adopt it have no exposure to vendor pricing changes, service discontinuation, or contract renegotiations. Its components can be replaced as models change: the pilot used GPT-4o, whereas the AHS deployment now mainly uses Claude models (Anthropic PBC), a change made through configuration rather than re-engineering. Long-term maintenance of the codebase depends on continued institutional and community contribution, and sustainability in other health systems has not been tested.

### Data Collection and Analysis

During the pilot, the system automatically recorded use and session metadata. A *session* was defined as the set of 1 or more recordings, transcripts, and generated notes associated with a single patient encounter. We summarized use descriptively. Data for the period after the pilot were obtained from the application’s administrative dashboard. Physicians were emailed an optional survey 3 months after they began using the tool. The survey was administered in REDCap (Vanderbilt University), and responses were accepted through December 31, 2025, extending beyond the pilot period. Respondents rated 10 statements about the tool on a 5-point scale (1=“not at all true,” 2=“somewhat true,” 3=“moderately true,” 4=“very true,” and 5=“completely true”). The statements covered ease of learning, available support, privacy and data security, the verbal consent process, long-term use, colleagues’ interest, documentation efficiency and quality, time with patients, and workflow integration. Respondents also rated overall user-friendliness on a 7-point scale from 1 (“worst imaginable”) to 7 (“best imaginable”), on which a score of 5 was labeled good, and estimated the time saved per shift in 15-minute bands. Responses were summarized as medians and interquartile ranges (IQR).

### Ethical Considerations

The study was approved by the Health Research Ethics Board (Health Panel), University of Alberta (Pro00138648) on July 11, 2024. Verbal consent was sought before recording (refer to the Data Governance section). AHS served as the data custodian for all clinical audio, transcripts, and generated notes, which were retained within the secure AHS environment. For this study, AHS performed analyses on aggregate operational metadata and provided the authors only with deidentified, aggregated results; no individual-level patient or clinician records were released to the research team. The user survey was conducted under a separate quality improvement approval (Pro00146623); consent was implied by completion, and the deidentified responses were stored and analyzed at the University of Alberta under a separate Research Ethics Board approval (Pro00153493).

## Implementation (Results)

### Coverage

Coverage was subnational, with implementation limited to 1 Canadian province (Alberta). Between November 2024 and July 2025, the system was used by 197 emergency physicians whose registered primary sites spanned 105 urban and rural facilities; however, the facility where each session took place was not recorded. These users represented 13% (197/1525) of the active emergency physicians in AHS. After the pilot, access was opened to other departments, including primary care and pediatrics. Approximately 1700 users had registered by August 31, 2026, and 738 (about 43%) had recorded a session in the preceding 30 days. By September 30, 2026, registrations had reached approximately 2000.

### Outcomes

During the pilot, the system was used for 21,548 sessions, and monthly session volume increased from 675 sessions in November 2024 to 5463 sessions in July 2025. Mean recorded session length was 7.6 minutes, yielding more than 2700 hours of clinical audio. Across all users, 42% (82/197) customized at least 1 documentation template. Operating costs averaged less than US $30 per physician per month. After the pilot, use continued to grow: monthly session volume reached approximately 25,000 sessions in August 2026, and by August 31, 2026, more than 157,000 sessions had been recorded. Mean recorded session length had risen to approximately 11 minutes, largely reflecting use outside emergency departments.

In the follow-up survey, between 69 and 73 physicians answered each item. Overall user-friendliness was rated as good (median 5, IQR 4-6), and the median self-reported time saved was 16 to 30 (IQR 0-15 to 46-60) minutes per shift. Agreement was highest for the statements regarding ease of learning, available support, privacy and data security, the verbal consent process, and long-term use (median 4; “very true”). Agreement was lower for statements regarding colleagues’ interest, documentation efficiency, and time with patients (median 3; “moderately true”), and lowest for workflow integration (median 2.5) and documentation quality (median 2; “somewhat true”).

### Lessons Learned

We built the first prototype of Berta with Gradio (Hugging Face Inc) [42], which allowed rapid development but did not handle long recordings reliably. We therefore rebuilt the front end in Next.js; starting with a production web framework would have avoided this rework. Beyond the software, adoption was supported by co-design with practicing emergency physicians, whose weekly feedback shaped the tool to everyday workflows, and by the ability to customize templates, prompts, and local terminology. Use grew without a mandate, although co-design required a sustained commitment of physician time that similar projects should plan for.

The pace of deployment, however, was set largely by governance and privacy review rather than by software development. Therefore, organizations planning a similar implementation should allow time and resources for these processes early. Implementation and maintenance of the AHS deployment have rested largely on 1 developer (JD), which enabled rapid iteration but creates risks related to continuity and support; similar projects should plan for shared code ownership. The deployment also depends on an enterprise data platform (Snowflake) that provided governed infrastructure but introduced platform-specific dependencies and costs. In addition, access restrictions meant that the platform could not be shared with the research team. Finally, enabling the open-source release, including the development of an intellectual property agreement, took considerable effort. Uptake is at an early stage: by October 2026, the repository had 26 stars and 7 forks, but we know of no sustained deployment by another organization and have received no external code contributions. Thus, reuse beyond the original setting remains untested.

## Discussion

### Principal Findings

This work demonstrates that AI-enabled clinical documentation can be deployed at a provincial scale within existing health system infrastructure without relying on commercial AI scribe vendors. In partnership with AHS, we developed an open-source AI scribe platform, and a related closed-source implementation has been used by 197 emergency physicians over 9 months. To our knowledge, Berta is the first open-source AI scribe platform released together with evidence from a related deployment at the health system scale. By releasing the underlying codebase, we provide a reproducible foundation for health systems seeking to evaluate or implement AI documentation tools within their own secure environments. The open-source release reduces the infrastructure and engineering effort required for adoption: the platform can be launched on Amazon Web Services from a prebuilt template or run on a single workstation [29]. The analysis presented in Multimedia Appendix 1 [29,30,38,43,44] provides an estimate of the number of physicians that could be supported by a server equipped with 8 NVIDIA A100 GPUs (NVIDIA Corp). Health systems that adopt Berta still need technical and governance capacity for privacy, security, and clinical oversight, and we have not tested the platform in resource-limited settings. Retention of data within institutional infrastructure also created opportunities that are unavailable when data reside on vendor servers: the clinical audio constitutes a potential asset for quality improvement and, subject to appropriate consent and ethics approval, for research. Audit trails also remained within institutional control, enabling end-to-end traceability in the event of clinical or legal review.

### Comparison With Prior Work

Most prior AI scribe deployments have used closed, vendor-hosted architectures. A recent in-house deployment built on local speech recognition and language model components reduced documentation time and improved patient engagement, supporting the use of in-house scribes where data privacy, cost, or localization are key concerns [45]. Our work extends this literature in 2 directions. First, it demonstrates that deployment at a comparable scale is achievable using an open, modular architecture hosted entirely within institutional infrastructure, addressing transparency and data sovereignty concerns raised in recent reviews [19-21,24]. Second, the observed operating costs (less than US $30 per physician per month, excluding personnel costs) are 70% to 95% lower than published commercial subscription benchmarks [22,23], providing an empirical reference point for health systems conducting procurement evaluations or considering in-house alternatives.

### Limitations

First, the AHS deployment does not integrate directly with the EHR because of an institutional restriction. Physicians must copy and paste generated notes into the EHR, introducing a manual step that commercial products with direct integration may avoid. Integration planning should therefore have begun earlier in the implementation process. Second, the results reported here are descriptive use and cost metrics from a single health system. We did not conduct a formal evaluation of note quality, documentation time, system reliability, or clinician burnout outcomes, and the study was not designed to make causal inferences about clinical impact. The cost reduction should therefore not be interpreted as evidence of superior value: a lower-cost system that produced inferior documentation could incur downstream costs through additional clinician editing, documentation errors or omissions, governance burden, or medicolegal exposure. Voluntary uptake, continued growth in use after the pilot, and respondents’ reported time savings and intention to keep using the scribe suggest that clinicians found it useful. However, these findings are not measures of documentation quality, which respondents rated lowest. The survey response rate was low. Finally, the operating cost estimate is derived from shared enterprise infrastructure that does not allow precise attribution of costs to individual users; actual costs will vary with scale, model selection, and the chosen deployment environment.

### Future Directions

Formal evaluation of the expanded deployment will assess note creation time, note quality, system reliability, and the number of patients seen per shift, comparing AI-generated notes against established documentation standards. A retrospective evaluation of documentation time is underway. Longer-term directions include integrating transcripts with EHRs to enable longitudinal retrieval and exploring richer contextual inputs (such as prior patient history) to improve note generation. Findings from the expanded AHS deployment will inform ongoing updates to the open-source codebase.

### Conclusions

AI-driven documentation tools are rapidly entering clinical practice, yet most available solutions operate as proprietary systems that limit organizational control over data, customization, and cost. This work provides evidence that an alternative model is viable: an in-house AI scribe that can be deployed at scale within existing health system infrastructure and operated at a fraction of the commercial operating cost. By releasing Berta publicly, we offer health systems a reproducible path to evaluate and deploy AI documentation technology on their own terms.

## Supplementary material

10.2196/105385 Multimedia Appendix 1 Load simulation of the transcription and note-generation services.

10.2196/105385 Checklist 1 iCHECK-DH checklist.

## Abbreviations

AHS: Alberta Health Services
ASR: automatic speech recognition
EHR: electronic health record
GPU: graphics processing unit
iCHECK-DH: Guidelines and Checklist for the Reporting on Digital Health Implementations
LLM: large language model
