# Supplementary material for: Berta, an Open-Source, Modular AI Scribe for Clinical Documentation: Implementation Report
Source: JMIR Med Inform. 2026 Nov 10;14:e105385. doi: 10.2196/105385 (PMC13750627; doi:10.2196/105385)
Supplement: Supplementary file 1 [file medinform-v14-e105385-s001.pdf]

Multimedia Appendix 2. Load simulation of the transcription and note-generation services

Manuscript: Berta, an Open-Source Modular AI Scribe for Clinical Documentation: Implementation Report.

Summary

In a 1-hour simulation on 1 virtual machine with 8 graphics processing units (GPUs), a WhisperX transcription service and a Llama 3.3 70B note-generation service returned 99.0% (1,639/1,655) of notes within 60 seconds for 550 simulated physicians, each starting an average of 3 encounters per hour. The 2 services set up for this test used the prompt and note template of the open-source Berta release. The results apply to this configuration only and do not describe the Alberta Health Services (AHS) deployment.

Introduction

Background

The main text does not report response times for the Alberta Health Services (AHS) deployment. Organizations planning their own deployment of the open-source release [1] need an estimate of how many physicians 1 virtual machine can support and how long a note takes to return.

Objective

This simulation estimated how many physicians, working at the same time, 1 virtual machine with 8 graphics processing units (GPUs) can support with WhisperX and Llama 3.3 70B, with at least 95% of notes returned within 60 seconds.

Methods

Design

This was a load simulation. A script submitted audio recordings to a transcription service and sent each transcript to a large language model (LLM) for note generation, at the rate expected from a set number of physicians working at the same time. It timed each step. A simulated visit was 1 recording processed in this way.

The simulation was run in September 2026 on 1 cloud virtual machine from Denvr Dataworks [2]. The script ran on the same virtual machine and called the 2 services directly.

Hardware and Software

Table S1 shows the configuration.

Table S1. Hardware and software configuration.

Component	Configuration
Hardware	8 NVIDIA A100 80GB (SXM) GPUs; 200 virtual central processing units; 940 GB of memory; 17 TB of solid-state storage
Transcription	WhisperX [3] with the large-v3 model on 4 GPUs; 16-bit floating point; batch size 16; word-level alignment; 2 concurrent jobs per GPU (8 slots in total)
Note generation	Llama 3.3 70B Instruct (Meta Platforms, Inc) served with vLLM 0.8.5 [4]; 2 model instances, each on 2 GPUs; 16-bit precision
Routing	Each recording went to the next free transcription slot; the script sent each transcript to the model instance with fewer requests in progress

GPU: graphics processing unit.

Relation to the Open-Source Release

The Berta web application was not run. The test used 2 software components that the release supports [1], WhisperX and vLLM, as stand-alone services. It used the release's system prompt, "Full Visit" note template, and request settings (temperature 0; maximum note length of 1,024 tokens).

The configuration in Table S1 was chosen for this test and differs from the example configurations in the release. There, WhisperX runs inside the web application on 1 device, with 32-bit floating point and without word-level alignment. The release's vLLM examples use other models (Llama 3.1 70B Instruct, Llama 3.1 8B Instruct, or a 4-bit version of Llama 3.3 70B). The web application also sends a second, short request to the LLM to label each transcript, which was not simulated.

Simulated Workload

Each simulated physician started an average of 3 encounters per hour. The number of simulated physicians is the number working at the same time, not the number of registered users. So 550 simulated physicians correspond to about 1,650 visits per hour, or 1 new visit about every 2.2 seconds.

Visits started at random times (a Poisson process). The times were generated from a fixed random seed for each load level, so the 2 runs at 600 simulated physicians had the same visit times.

Each visit submitted 1 of 100 audio recordings of simulated patient-physician interviews from an open dataset of 272 recordings [5]. The 100 recordings were 46 musculoskeletal, 41 respiratory, 6 gastrointestinal, 5 cardiac, 1 dermatological, and 1 other case, and were 6.6 to 18.6 minutes long (mean 12.5 minutes). They were used in random order, and the order was reshuffled after all 100 had been used. A recording that was still being processed was not given to another visit.

The transcript was sent to the LLM with the system prompt and "Full Visit" note template, at temperature 0 and a maximum note length of 1,024 tokens. This fixed part of each prompt, with chat formatting, was 828 tokens. Prompts had a median length of about 2,600 tokens, and notes about 490 tokens.

Each request carried a unique tag, so that the LLM could not reuse cached computation for a repeated recording. About 31% of prompt tokens were served from cache, which matches the fixed part of the prompt.

Procedure

Each run followed the same steps:

1. The number of simulated physicians was set (eg, 550). The script then started visits at random times for the length of the run (10 minutes or 1 hour).
2. When a visit started, the script submitted the next recording to the transcription service. Response time was counted from this moment.
3. The recording was transcribed. It first waited in a queue if all 8 transcription slots were busy.
4. The script sent the transcript to the LLM with the system prompt and note template, and the LLM generated the note.
5. Response time ended when the note was complete. The script saved the time taken by each step.
6. When the length of the run was reached, no new visits were started. Visits in progress were followed until their notes were complete.

Load Levels

Load was set by the number of simulated physicians. Runs of 10 minutes at 400, 500, 600, and 700 simulated physicians were used to find the range in which the criterion for a sustainable load (see Outcome Measures) stopped being met. A run at 800 simulated physicians was started and then stopped, because the criterion had already not been met at 700. It gave no results.

The limit was then tested in 1-hour runs, first at 600 and then at 550 simulated physicians. Testing stopped at the first level that met the criterion. The result for each load level and run length comes from a single run.

Outcome Measures

The main measure was response time: the time from submission of a recording to completion of the note, including time waiting in a queue. It excludes the time to upload audio from a physician's device.

For each visit, the script also recorded transcription time (including time waiting for a free slot), note-generation time, whether the visit ended in an error, and whether the note reached the length limit. The number of requests waiting for each service was sampled about every 5 seconds.

A load level was counted as sustainable if at least 95% of visits finished within 60 seconds, no visit ended in an error, and the number of waiting requests did not keep rising during the run.

Data Analysis

Results are summarized with descriptive statistics (counts, percentages, medians, 95th percentiles, and ranges). Percentiles were calculated with linear interpolation. All visits that started during a run were included. No statistical tests were done.

Ethical Considerations

No patient data and no data from the AHS deployment were used. The recordings are publicly available simulated interviews. In each, the patient and the physician were played by senior medical students or resident physicians [5].

Results

Overview

The 6 runs included 4,633 simulated visits (Table S2). Each of the 100 recordings was used 44 to 48 times. In these runs, no visit ended in an error and no note reached the length limit. In the 1-hour run at 550 simulated physicians, 1 request to the LLM was retried after a connection error and then completed.

One-Hour Run at 550 Simulated Physicians

Over 60 minutes, 1,655 visits were started (1,650 expected), and 99.0% (1,639/1,655) of notes were returned within 60 seconds. The median response time was 44.1 seconds, the 95th percentile was 55.1 seconds, and the slowest note took 66.0 seconds.

In each 10-minute period of the hour, 98.2% to 100% of notes were returned within 60 seconds. At most 8 recordings were waiting for transcription at any sampled time, and this number did not rise over the hour. The criterion for a sustainable load was met.

Response time increased with recording length. For recordings shorter than 13 minutes, 13 to less than 16 minutes, and 16 minutes or longer, the median response times were 40.9, 48.2, and 52.1 seconds. The shares returned within 60 seconds were 100% (959/959), 98.3% (572/582), and 94.7% (108/114). All 16 notes that took longer than 60 seconds had also waited for a free transcription slot (median wait 7.1 seconds).

One-Hour Run at 600 Simulated Physicians

Over 60 minutes, 1,803 visits were started (1,800 expected), and 88.1% (1,589/1,803) of notes were returned within 60 seconds, so the criterion was not met. Across the 10-minute periods of the hour, this share ranged from 60.0% to 99.7%.

Most notes that took longer than 60 seconds (192/214, 90%) were for visits that started between minutes 23 and 33 or between minutes 48 and 53 of the run. Each of these periods included a burst of visits: up to 94 and 85

visits started within 2 minutes, where 60 were expected. Outside these periods, 98.3% (1,259/1,281) of notes were returned within 60 seconds.

All 214 notes that took longer than 60 seconds had waited for a free transcription slot (median wait 17.9 seconds). Across all visits, the 95th percentile wait was 19.6 seconds, compared with 6.4 seconds at 550 simulated physicians. Up to 21 recordings were waiting at a sampled time, but the number did not keep rising: it averaged 2.3 in each half of the hour.

Ten-Minute Runs

The criterion was met at 400, 500, and 600 simulated physicians and not at 700 (Table S2). At 700, the number of recordings waiting for transcription rose during the run.

Because visits started at random times, these short runs had 6% fewer to 12% more visits than expected. The runs at 500 and 600 simulated physicians had almost the same number of visits (281 and 282). The 10-minute run at 600 also had the same visit times as the first 10 minutes of the 1-hour run at 600. The 10-minute runs served to choose the levels for the 1-hour runs.

Note Generation Under Load

No request was waiting for the LLM at any sampled time in any run. Under higher load, note generation became slower instead: its median time rose from 28.9 seconds at 400 simulated physicians to 31.7 seconds at 700 (Table S2).

Table S2. Response times by load level.

Run length	Simulated physicians	Visits expected, n	Visits started, n	Notes returned within 60 seconds, n (%)	Response time, median (seconds)	Response time, 95th percentile (seconds)	Transcription, median (seconds)	Note generation, median (seconds)
10 minutes	400	200	220	220 (100)	41.4	51.7	12.3	28.9
10 minutes	500	250	281	276 (98.2)	44.2	57.3	13.9	30.0
10 minutes	600	300	282	281 (99.6)	43.9	54.2	13.4	30.4
10 minutes	700	350	392	155 (39.5)	64.3	97.1	30.5	31.7
1 hour	550	1,650	1,655	1,639 (99.0)	44.1	55.1	13.6	30.1
1 hour	600	1,800	1,803	1,589 (88.1)	47.3	66.7	15.8	30.7

Transcription time includes time waiting for a free slot. Expected visits are 3 per simulated physician per hour.

Discussion

Principal Findings

In a 1-hour run on 1 virtual machine with 8 GPUs, WhisperX and Llama 3.3 70B returned 99.0% of notes within 60 seconds for 550 simulated physicians working at the same time, or about 1,650 visits per hour. In the 1-hour run at 600 simulated physicians, the 95% criterion was not met.

In the 1-hour runs, every note that took longer than 60 seconds had waited for a transcription slot. At 600 simulated physicians, most of these notes came from 2 periods with bursts of visits. Note generation took a median of about 30 seconds at every load level, longer than transcription. Response time also increased with recording length.

These figures give organizations a reference point for sizing their own deployment.

Limitations

- The results come from 1 hardware and software configuration that was set up for this test. It differs from the example configurations of the open-source release (see Relation to the Open-Source Release).
- The result for each load level comes from a single run with 1 set of random visit times, so the level at which the criterion stops being met is approximate. Levels between 550 and 600 simulated physicians and runs longer than 1 hour were not tested.

- The visit rate (3 visits per physician per hour) was an assumption. The recordings were 6.6 to 18.6 minutes long, and longer recordings are likely to take longer than reported here.
- Only processing on the virtual machine was simulated, and the script that generated the load ran on the same virtual machine. The web application, authentication, storage, and upload of audio over a network were not included.
- Transcription accuracy and note quality were not assessed.
- The AHS deployment used a different LLM and infrastructure, and its response times are not reported here.

Conclusions

In this simulation, 1 virtual machine with 8 GPUs returned 99.0% of notes within 60 seconds for 550 simulated physicians, each starting an average of 3 visits per hour. The result comes from a single 1-hour run on the tested configuration and does not describe the AHS deployment.

References

1. Vaid S, Weldon M, Dunn J, et al. Berta: an open-source, modular tool for AI-enabled clinical documentation. GitHub. 2025. https://github.com/phairlab/berta-ai-scribe [accessed Oct 5, 2026]
2. Denvr Dataworks. Denvr: AI platform services. https://www.denvr.com/ [accessed Oct 5, 2026]
3. Bain M, Huh J, Han T, Zisserman A. WhisperX: time-accurate speech transcription of long-form audio. arXiv. Preprint posted online March 1, 2023. doi:10.48550/arXiv.2303.00747
4. Kwon W, Li Z, Zhuang S, et al. Efficient memory management for large language model serving with PagedAttention. arXiv. Preprint posted online September 12, 2023. doi:10.48550/arXiv.2309.06180
5. Fareez F, Parikh T, Wavell C, et al. A dataset of simulated patient-physician medical interviews with a focus on respiratory cases. Sci Data. 2022;9(1):313. PMID:35710769 doi:10.1038/s41597-022-01423-1
