# Supplementary material for: Berta, an Open-Source, Modular AI Scribe for Clinical Documentation: Implementation Report
Source: JMIR Med Inform. 2026 Nov 10;14:e105385. doi: 10.2196/105385 (PMC13750627; doi:10.2196/105385)
Supplement: Supplementary file 2 [file medinform-v14-e105385-s002.pdf]

Multimedia Appendix 1. Checklist of iCHECK-DH guidelines. iCHECK-DH: Guidelines and Checklist for the Reporting on Digital Health Implementations. Manuscript: Berta, an Open-Source Modular AI Scribe for Clinical Documentation: Implementation Report. The last column summarizes how each item is addressed and gives the manuscript sections where it is reported. AHS: Alberta Health Services; AI: artificial intelligence.

SECTION		ITEM	DESCRIPTION	HOW THE ITEM IS ADDRESSED (AND WHERE IT IS REPORTED)
**TITLE**	1	Title (M[1])	Identification as an implementation report, and description of the implementation in the title and/or keywords	The title identifies the article as an implementation report and names the intervention: "Berta, an Open-Source Modular AI Scribe for Clinical Documentation: Implementation Report." The keywords include ambient AI scribe, clinical documentation, and emergency medicine.<br>*Reported in: Title; Keywords*
**ABSTRACT**	2	Abstract (M)	Provide a summary of the key elements of the implementation report, including a description of the implementation strategy, the intervention, defining the key elements of the implementation and health outcomes and specify the key KPIs/Outputs. We recommend describing the main aspects of the research in the following order: Background - Objectives - Methods - Implementation (Results) - Conclusions - (Optional: Trial Registration).	The abstract is structured as Background, Objective, Methods, Implementation (Results), and Conclusions. It describes the intervention (an open-source AI scribe), how it was implemented (a closed-source deployment in AHS, developed alongside Berta, used by emergency physicians and later opened to other departments), and the key outputs: users, facilities, sessions, hours of audio, monthly volume, session length, template customization, and operating cost per physician per month. No clinical outcomes are reported; the abstract concludes that full cost of ownership and documentation quality require further evaluation.<br>*Reported in: Abstract*
**INTRODUCTION**	3	Context (M)	Describe the geographical areas, organizations, target populations and implementation context. Consider social, cultural, economic, political, health care and organizational barriers, infrastructures and facilitators that may influence implementation elsewhere. Explicitly highlight whether a national digital health strategy exists and whether implementation is aligned with the strategy.<br><br>Describe the stage of the implementation (Developing or Adapting Solution / Piloting and Evidence generation / Package and Advocacy /Acceleration / Deploying / Scaling up / Hand over or Complete).[2]	The implementation took place in Alberta, Canada, within AHS, which at the time was Canada's largest provincially integrated health system, providing publicly funded care to approximately 5 million residents in urban centers and dispersed rural communities. Clinical documentation is recorded in Connect Care, a provincewide electronic health record (Epic Systems); the AI scribe was not integrated with it. The target population was emergency physicians. The implementation was not part of a national program; whether a national digital health strategy exists, and whether the implementation is aligned with one, is not reported. The implementation progressed from piloting and evidence generation (November 2024 to July 2025) to an approved provincial scale-up (scaling up). Facilitators (co-design with emergency physicians and local customization) and barriers (the time required for governance and privacy review, and an institutional restriction on integration

[1] M: Mandatory item

[2] *Stages adapted from WHO Digital Health Atlas*

			with the electronic health record) are described in Lessons Learned and Limitations. *Reported in: Introduction, Context; Methods, Pilot Implementation; Implementation (Results), Lessons Learned; Discussion, Limitations*
4	Problem statement (M)	Description of the health care or public health problem, challenge, or deficiency that the implementation aims to address. (If applicable, include a reference to the 'health system challenge' of the WHO Classification of Digital Health Interventions[3] in the description)	Clinical documentation places a heavy burden on physicians: they spend nearly 2 hours on electronic health records and desk work for every hour of direct patient care, and documentation demands are associated with burnout, reduced job satisfaction, and attrition. The burden is particularly heavy in high-acuity settings such as emergency departments, where the workforce is already under strain and time-sensitive clinical decisions compete with the need for prompt, thorough records. *Reported in: Introduction, Problem Statement*
5	Similar Interventions (M)	Mention whether this implementation was inspired by another existing one, and if so, what is the added value of your intervention, if any, compared to the initial one? And what, if anything, has been done differently?	Berta was developed independently of commercial ambient AI scribes, as an open, modular alternative that can be deployed within an institution's own infrastructure. Commercial products often process data on vendor-controlled infrastructure, and even when hosted locally, their models, prompts, and updates remain closed to independent audit. Berta can be audited and customized locally, and the operating costs of the AHS deployment were 70% to 95% below published commercial subscription prices, excluding personnel costs. Compared with a recent in-house AI scribe deployment, this work shows that equivalent-scale deployment is achievable with an open, modular architecture hosted entirely within institutional infrastructure. *Reported in: Introduction, Similar Interventions; Discussion, Comparison With Prior Work*

[3] https://apps.who.int/iris/bitstream/handle/10665/260480/WHO-RHR-18.06-eng.pdf?sequence=1&isAllowed=y

**METHODS**	6	Aims and Objectives (M)	Describe the main objectives and the overall aim of the implementation. Describe how these will be measured using predefined primary and secondary outcome(s) and key performance indicators for this implementation and the expected intervention(s).<br>*For example: indicators or proxy-indicators measuring direct health outcomes (e.g., HbA1c for diabetic patients); Key Performance Indicators (e.g., number of users, number of users that are properly trained, user satisfaction); Indicator assessing a particular process (e.g., administrative time for patient admission);*<br>(If there was no evaluation, provide detailed explanation for reasoning)	The aim was to develop an open-source, modular AI scribe that can be deployed within institutional infrastructure and to evaluate its real-world use across a provincial health system. This was a descriptive implementation study. The key performance indicators were session counts, unique users, audio duration, template customization, monthly utilization, and operating cost per physician per month; an optional user survey also measured user-friendliness, time saved per shift, and agreement with 10 statements about the tool. Clinical outcomes were outside the scope of the pilot; a formal evaluation of the expanded deployment is planned, and a retrospective evaluation of documentation time is underway.<br>*Reported in: Abstract, Objective; Methods, Aims and Objectives and Data Collection and Analysis; Discussion, Future Directions*
	7	Blueprint summary (M)	Describe the design and key features of the intervention and key points of the implementation strategy and roadmap.	Berta was co-designed with emergency physicians through weekly meetings, with core development between March and November 2024. In routine use, clinicians record or upload audio of a patient encounter in a desktop or mobile browser; the system transcribes it with a speech recognition model and uses a large language model to generate a draft note from configurable or custom templates, which the clinician reviews, edits, and copies into the electronic health record. A closed-source implementation developed alongside Berta was deployed at AHS in November 2024. Physicians were given access individually after registration and brief online training; adoption was voluntary and spread by word of mouth, and after early adopters, physicians at further emergency departments were invited in weekly waves between April and June 2025. After the pilot, access was opened to other departments.<br>*Reported in: Methods, Co-Design and Development, Technical Architecture (Figures 1 and 2), and Pilot Implementation; Implementation (Results), Coverage*
	8	Technical Design (M)	Reasons for developing or choosing this tool. Does it combine several tools? Provide a brief description of the tool(s) (functionality and architecture) and how it fits into the health enterprise architecture and investment roadmap (if applicable). Indicate whether the solution is based on an existing solution or has been developed or purchased specifically for this intervention.<br>Describe the type of technology used (e.g., AI applications), license of the technology (open source, free, commercial, IP ownership etc.), include code documentation (if available), link to the application, link to wiki or project website.	Berta was developed as an open alternative to commercial AI scribes that can run within institutional infrastructure. It combines a Next.js front end and a FastAPI backend with interchangeable speech recognition and large language model components, and can run on anything from a single workstation to a cloud environment. The AHS deployment (internally called "Jenkins" or the "AHS Digital Scribe") is a closed-source implementation developed in parallel with the open-source Berta code base by overlapping teams, sharing design, functionality, and portions of code but maintained separately: the pilot ran within AHS's existing Snowflake AI Data Cloud infrastructure, used WhisperX for transcription and GPT-4o for note generation, and was not integrated with the electronic health record. The open-

			source Berta code base is released under the Apache 2.0 license on GitHub (https://github.com/phairlab/berta-ai-scribe). A load simulation of transcription and note generation on 1 virtual machine is reported in Multimedia Appendix 2.<br>*Reported in: Introduction, Similar Interventions; Methods, Technical Architecture (Figures 1 and 2, Table 1) and Pilot Implementation; Discussion, Principal Findings; Data Availability; Multimedia Appendix 2*
9	Target (M)	The target refers to the focus or recipient of the intervention. It is the specific person, group, system, or problem that the intervention aims to change or improve. The characteristics of the targeted "site(s)" (locations, staff, resources, etc.) for implementation and any eligibility criteria. The population targeted by the intervention and any eligibility criteria.	The target was emergency physicians in AHS. Access was granted to individual physicians, not sites, after registration and brief online training, and adoption was voluntary. During the pilot, 197 emergency physicians used the system; their registered primary sites spanned 105 urban and rural facilities.<br>*Reported in: Methods, Pilot Implementation; Implementation (Results), Coverage*
10	Data (M)	Describe the data governance, including life cycle (collection, processing, storage, modification, sharing, suppression), the data ownership (mention whether patients actually have access to the data), data protection measures, confidential use of routine data, expected level of data integration, data for research, cross-border data agreement, if any, the applicable legal framework, and how the project complies with it. Data consent: Has patient consent been obtained? Describe the approach to data protection and cybersecurity (e.g. security by design, privacy by design, etc.) and where the data is hosted. (e.g., in-country, cloud based, hybrid model etc.). Describe, if applicable, the government preferences in terms of data policies.	Physicians were instructed to obtain patients' verbal consent before recording, using a standard script. The pilot ran in AHS's Snowflake AI Data Cloud environment, and all clinical audio and text remained within the AHS firewall and were not exported. Physicians can delete recordings (3,677 of 21,548 pilot sessions [17%] were deleted); retained recordings are held by AHS, as data custodian, under the same provincial health information legislation as other clinical records and are kept for the duration of the project. The pace of deployment was set largely by governance and privacy review; Berta can be deployed within a secure virtual private cloud with multiple user authentication systems, and audit trails remained within institutional control. The research team received only de-identified, aggregated results of the usage data, and individual-level clinical data are not publicly available; research use of the audio would require appropriate consent and ethics approval. Not reported: patient access to recordings and generated notes, the geographic location of the hosting environment, any cross-border data agreement, and government data policy preferences.<br>*Reported in: Methods, Technical Architecture (Figure 1), Pilot Implementation, Data Governance, and Ethical Considerations; Implementation (Results), Lessons Learned; Discussion, Principal Findings; Data Availability*
11	Interoperability (M)	Describe the interfaces (what other systems does the tool connect to) and the standards that were used (which specific ones and rationale of choice) (e.g., semantic ontologies such ICD as SNOMED, LOINC or technical standards such as HL7 FHIR, etc.).	The AI scribe was not integrated with the electronic health record because of an institutional restriction; physicians transferred notes by copy and paste. No clinical terminology or data exchange standards (eg, Fast Healthcare Interoperability Resources) were used.

			Standards-based integration with the electronic health record is planned.<br>*Reported in: Methods, Interoperability; Discussion, Limitations*
12	Participating entities (M)	Describe the implementing organization(s): Type of organisation(s), mission, leadership, vision, etc.<br><br>Government involvement: Describe whether the government was involved in the implementation, at what level and at what stage(s).<br><br>Partners: Describe all partners (organisations) and their role in the implementation.<br><br>Funders: List all actors and stakeholders who have funded or invested in the development of the implementation (if different from the implementation, e.g. using an existing digital health intervention). Indicate their level of involvement in terms of funding.<br><br>Mention which entity will own the final product and intellectual property after the implementation phase.	Berta was developed by a University of Alberta team in partnership with AHS, the provincial health authority, which owns and operates the deployed application (Jenkins), served as data custodian, and analyzed the aggregate operational metadata. The co-design team comprised 4 emergency physicians, 2 machine learning engineers, 1 project analyst, and 2 supervising professors. The project was funded through a 2-year Health Care Unburdened grant of CAD $1 million from the Canadian Medical Association, MD Financial Management, and Scotiabank; other supporters are listed in the Acknowledgments. The open-source Berta code base is released under the Apache 2.0 license. Not reported: the mission and vision of the implementing organizations, government involvement beyond AHS as the provincial health authority, and ownership of intellectual property in the open-source code base.<br>*Reported in: Introduction, Context; Methods, Co-Design and Development, Participating Entities, Costs, and Ethical Considerations; Funding; Acknowledgments; Data Availability*
13	Budget Planning (M)	Describe the planned budget for implementation (include costs such as change management, user training, project management, technology pricing, total cost of ownership). If possible, include actual costs, otherwise describe the range or percentage of the total budget. Indicate the period covered by the budget. Describe the budget for the intervention (e.g. development, purchase or adaptation of a free tool); if possible include real costs, otherwise describe as a percentage of the total budget. Indicate the duration covered by the budget.	The project was supported by a 2-year Health Care Unburdened grant of CAD $1 million. Core development took approximately 13 person-months of engineering effort (1 full-time and 1 part-time machine learning engineer) between March and November 2024. Operating costs during the pilot (November 2024 to July 2025) were estimated from Snowflake service consumption (servers, large language model tokens, and storage) and averaged less than US $30 per physician per month. These estimates exclude development labor and ongoing personnel costs; maintenance after deployment was absorbed within AHS operations and was not tracked separately. Not reported: a breakdown of the grant budget by cost category (eg, change management, user training, project management, technology) and total cost of ownership.<br>*Reported in: Abstract, Methods; Methods, Co-Design and Development, Costs, and Sustainability; Implementation (Results), Outcomes*
14	Sustainability (M)	Describe the Business model including the sustainability model (financial, economic, environment etc.). If possible, put outcomes in relation to cost to assess sustainability. Describe long term exit strategies, and all dimensions considered to sustain the project after the end of funding. If applicable, describe potential institutionalization of the project.	For the AHS deployment, access was opened to other departments after the pilot, institutionalizing the tool; maintenance and upgrades are carried out by 1 developer with support from existing AHS information technology staff, and these costs are absorbed within AHS operations. The deployment depends on an enterprise data

				platform (Snowflake), which introduces platform-specific dependencies and costs. Use continued to grow after the pilot. Operating costs during the pilot averaged less than US $30 per physician per month, excluding personnel costs. The open-source platform is released under the Apache 2.0 license, so adopters are not exposed to vendor pricing changes or service discontinuation, and its components can be replaced as models change (the pilot used GPT-4o, whereas the AHS deployment now mainly uses Claude models, a change made through configuration). Long-term maintenance depends on institutional and community contribution, sustainability in other health systems has not been tested, and findings from the expanded deployment will inform updates to the open-source code base. Not reported: a formal exit strategy for the end of grant funding, and environmental sustainability.<br>*Reported in: Methods, Sustainability; Implementation (Results), Outcomes; Discussion, Limitations and Future Directions*
**RESULTS**	15	Coverage (M)	Describe whether the coverage of implementation is international, national, regional or at the level of e.g. municipalities. If coverage is sub-national, describe the regions. Provide information on the relative importance of the coverage (e.g. % of eligible population covered).	Coverage was subnational, limited to one Canadian province (Alberta). During the pilot, 197 emergency physicians used the system, representing 13% (197/1,525) of active emergency physicians in AHS; their registered primary sites spanned 105 urban and rural facilities. After the pilot, access was opened to other departments, including primary care and pediatrics, and approximately 2,000 users had registered by September 30, 2026.<br>*Reported in: Implementation (Results), Coverage*
	16	Outcomes (M)	Primary and other outcome(s) of the implementation. Detail the actual outcomes, using the pre-defined outcome measures (if applicable).	During the pilot, the system was used for 21,548 sessions, and monthly volume rose from 675 sessions in November 2024 to 5,463 in July 2025. Mean recorded session length was 7.6 minutes, yielding more than 2,700 hours of audio; 42% (82/197) of users customized at least one template; and operating costs averaged less than US $30 per physician per month. After the pilot, use continued to grow, with monthly volume reaching about 25,000 sessions in August 2026. In the follow-up survey (69 to 73 respondents per item), median user-friendliness was 5 of 7 (good), median self-reported time saved was 16 to 30 minutes per shift, and agreement was highest for ease of learning, available support, privacy and data security, the verbal consent process, and long-term use (median 4) and lowest for workflow integration (median 2.5) and documentation quality (median 2).<br>*Reported in: Implementation (Results), Coverage and Outcomes*
			Describe any lessons learned from the implementation experience that could be	The main challenges were that the first prototype, built with Gradio,

	17	Lessons learned (M)	used to improve future outcomes. This could include, but is not limited to, success factors, implementation challenges or budget considerations.<br><br>Success factors: Describe factors that positively influenced the implementation (e.g. involvement of key stakeholders). Also describe contextual factors that may have positively influenced the results (e.g. new legal requirements that facilitated adoption).<br><br>Challenges to implementation: Describe challenges (process-related, such as resistance to change, but also technical). Include contextual factors that may have affected the achievement of outcomes such as an unexpected change of government, or 'opposing key players' who, despite potential participation, may hinder implementation (e.g. software companies managing regional digital health may act as barriers to innovation).<br><br>Budget: Describe whether the implementation budget was adhered to, and if not, why not. Also detail the expected operational costs (e.g. licence, maintenance, human resources, updates to in-house developments) to estimate the total cost of ownership. Include real costs, otherwise describe them as a percentage of the total budget.<br><br>What recommendations can be drawn from the lessons learned?	did not handle long recordings reliably, so the front end was rebuilt as a native Next.js application; that the pace of deployment was set largely by governance and privacy review rather than by software development; that implementation and maintenance of the AHS deployment rested largely on 1 developer; that the deployment depends on an enterprise data platform that could not be shared with the research team; and that an institutional restriction prevented integration with the electronic health record. Success factors were co-design with practicing emergency physicians and the ability to customize templates, prompts, and local terminology; use grew without a mandate. Recommendations for similar projects are to start with a production web framework, plan for the physician time that co-design requires, allow time and resources for governance and privacy review early, plan integration from the outset, and plan for shared code ownership. Operating costs averaged less than US $30 per physician per month, excluding personnel costs; maintenance and upgrades are absorbed within AHS operations and were not tracked separately, so full cost of ownership requires further evaluation. Enabling the open-source release took considerable effort, and uptake is at an early stage (26 stars and 7 forks by October 2026; no known sustained deployment by another organization; no external code contributions), so reuse beyond the original setting remains untested. Not reported: whether the implementation budget was adhered to.<br>*Reported in: Implementation (Results), Lessons Learned and Outcomes; Methods, Costs and Sustainability; Discussion, Limitations; Abstract, Conclusions*
	18	Unintended consequences (NM[4])	Describe unintended consequences (positive or negative), harms or negative side-effects (if any).	Not reported (non-mandatory item).
**DISCUSSION**	19	Conclusion (M)	Summary of the conclusions and future implications.	An AI scribe developed in-house was deployed at scale within existing health system infrastructure at a fraction of the commercial operating cost; full cost of ownership and documentation quality require further evaluation. By releasing Berta publicly, the authors offer health systems a reproducible path to evaluate and deploy AI documentation technology on their own terms. Future work includes a formal evaluation of the expanded deployment, integration with the electronic health record, and updates to the open-source code base.<br>*Reported in: Abstract, Conclusions; Discussion, Principal Findings,*

[4] NM : Non-mandatory item

				Limitations, Future Directions, and Conclusions
**GENERAL**	20	General (NM)	If applicable, include statement(s) on regulatory approvals (including, as appropriate, ethical approval, governance approval), trial or study registration (availability of protocol), and conflicts of interest. For implementation reports with a research component, ethical approval or a waiver from an appropriate ethics committee is required. For those without a research component, ethical considerations may still be relevant, but do not necessarily require approval or waiver. Authors may consult this article[5] for further guidance on ethical considerations in their specific context"	The study was approved by the Health Research Ethics Board (Health Panel), University of Alberta (Pro00138648; July 11, 2024). The user survey was conducted under a separate quality improvement approval (Pro00146623), and de-identified responses were analyzed under Pro00153493. The pace of deployment was set largely by governance and privacy review. This was a descriptive implementation study, so trial registration does not apply. The authors declare no conflicts of interest, and the funders had no role in the study design, data analysis, writing, or decision to submit.<br>*Reported in: Methods, Aims and Objectives and Ethical Considerations; Implementation (Results), Lessons Learned; Funding; Conflicts of Interest*

[5] Eccles, M. P., Weijer, C., & Mittman, B. (2011). Requirements for ethics committee review for studies submitted to Implementation Science. Implementation science, 6(1), 1-3.
